# Origin of the light-induced spin currents in heavy metal/magnetic insulator bilayers

**DOI:** 10.1038/s41467-024-48710-6

**Published:** 2024-05-22

**Authors:** Hongru Wang, Jing Meng, Jianjun Lin, Bin Xu, Hai Ma, Yucheng Kan, Rui Chen, Lujun Huang, Ye Chen, Fangyu Yue, Chun-Gang Duan, Junhao Chu, Lin Sun

**Affiliations:** 1 Key Laboratory of Polar Materials and Devices (MOE), Department of Electronics, East China Normal University, Shanghai, China; 2 The Extreme Optoelectromechamics Laboratory (XXL), School of Physics and Electronic Science, East China Normal University, Shanghai, China; 3 Institute of Optoelectronics, Fudan University, Shanghai, China

**Keywords:** Spintronics, Electronic and spintronic devices, Surfaces, interfaces and thin films

## Abstract

Light-induced spin currents with the faster response is essential for the more efficient information transmission and processing. Herein, we systematically explore the effect of light illumination energy and direction on the light-induced spin currents in the W/Y_3_Fe_5_O_12_ heterojunction. Light-induced spin currents can be clearly categorized into two types. One is excited by the low light intensity, which mainly involves the photo-generated spin current from spin photovoltaic effect. The other is caused by the high light intensity, which is the light-thermally induced spin current and mainly excited by spin Seebeck effect. Under low light-intensity illumination, light-thermally induced temperature gradient is very small so that spin Seebeck effect can be neglected. Furthermore, the mechanism on spin photovoltaic effect is fully elucidated, where the photo-generated spin current in Y_3_Fe_5_O_12_ mainly originates from the process of spin precession induced by photons. These findings provide some deep insights into the origin of light-induced spin current.

## Abstract (web summary)

The authors study the light-induced spin current observed in W/Y_3_Fe_5_O_12_ heterojunctions, elucidating the photo-generated spin current, rather than light-thermally induced spin current, by photon-magnon interaction.

## Introduction

The exploration of spintronics has been advanced towards the manipulation of a pure spin current without a charge current. Due to the attributes of the larger angular momentum and zero charge current, pure spin current with much reduced Joule heating and power consumption is beneficial for information transport and processing^1–3^. Spin current is typically generated at a nonmagnetic heavy metal (HM) with strong spin-orbit coupling and a magnetic materials bilayered structure by physical phenomena such as the spin Hall effect (SHE)^4,5^, spin pumping effect (SPE)^6,7^, spin Seebeck effect (SSE)^8,9^, and spin photovoltaic effect (SPVE)^1,10^.

SPVE has attracted attention due to its ability to be excited by localized light and its faster response speed. In previous research, spin-polarized carriers were generated in the GaAs layer through the irradiation of circularly polarized light, and pure spin current was generated at the Pt/GaAs interface^11^. Recently, relevant works show that pure spin current can also be achieved at the Pt/Y_3_Fe_5_O_12_ (YIG) interface using unpolarized light^1^. This SPVE can be generated by photo-excitation of carriers near the interface and exists for light in the visible range^1,10,12^. Another work indicates that light-thermally induced temperature gradients (i.e., SSE) can excite magnons in YIG. Furthermore, the long-wavelength light causes the Fe^3+^ ions in the octahedra FeO_6_ from a high-spin state to a low-spin state, which increases the magnetization of YIG and subsequently enhances the magnon diffusion length^13^. In addition, a solution that separates the interface temperature gradient and bulk temperature gradients has been provided to analyze their contributions to spin currents^14^. For the light-induced spin current, most previous studies have focused on the generation and detection of magnons in YIG. The origin of light-induced spin current in the YIG system is still controversial. Therefore, it is worth exploring whether this light-induced spin current comes from SPVE or SSE.

Additionally, the mechanism of SPVE, i.e., the generation and transport of photo-excited spin current, has not been fully elucidated. Theoretically, SPVE is regarded as the bulk photovoltaic effect and its physical mechanism is similar to the “shift current” in nonlinear optical effects where the broken inversion symmetry (***P***) of the lattice is required^3,15,16^. Experimentally, SPVE induced by white light is believed to be associated with the photo-excitation of carriers in the proximized layer of HM, where the holes with a longer spin mean free path generate a much larger spin current than the electrons in the unoccupied bands, which leads to a net spin current *J*_net_ = *J*_e_ - *J*_h_^1,10,12^. Alternatively, there has been reported a spin convertance effect at the HM/magnetic insulator (FI) structure, which indicates that magnon current (i.e., spin wave) can be converted into spin current at HM/FI interface, and vice versa^4,17^. If the magnon current in the FI can be excited by light, the spin current is also obtained at the HM/FI structure by the spin convertance effect. This possibly implies that SPVE not only occurs in the proximity layer of HM, but also exists in FI. However, the experimental research on it is still lacking, and thus a comprehensive model is needed to explain the generation and transport mechanism of photo-excited spin currents.

In this work, we have successfully prepared the high-quality epitaxial W/YIG (111) heterostructure. Under illumination, inverse spin Hall voltage (*V*_ISHE_) observed in the W layer of this heterostructure provides evidence for the existence of a light-induced spin current. With the combination of experimental observation and heat transfer simulation, we confirm that the generation of spin currents induced by low light intensity is dominated by SPVE, whereas one caused by high light intensity is mainly excited by SSE. The microscopic model of SPVE in the W/YIG (111) bilayers is illustrated by means of photon-magnon interaction.

## Results

### Properties of W/YIG films

**Fig. 1:**
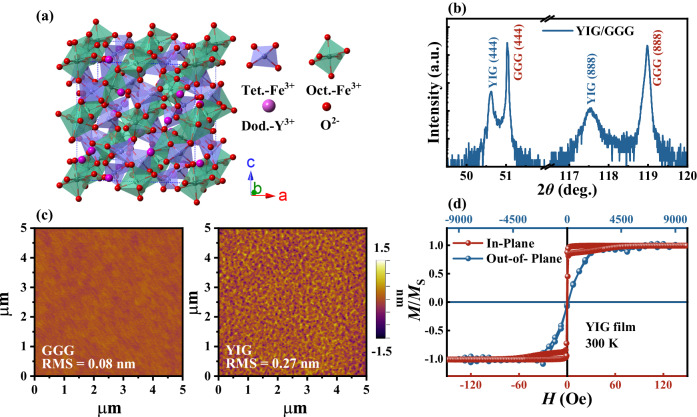
Properties of YIG/GGG films. **a** Crystal structure of YIG. The blue, green, purple, and red spheres represent the Tet.-Fe^3+^, Oct.-Fe^3+^, Dod.-Y^3+^ and O^2-^ ions, respectively. **b** Representative *θ*−2*θ* XRD pattern for a YIG (180 nm)/GGG film. **c** AFM images of the processed GGG substrate and a representative YIG (180 nm)/GGG heterostructure. **d** Normalized hysteresis loops measured with the magnetic field along the in-plane and out-of-plane at 300 K, respectively. *M*s is the saturation magnetization.

The crystal structure of YIG is a cubic crystal structure with space group *Ia-3d* and contains eight formula units (160 atoms), as shown in Fig. 1a. YIG consists of three sublattices, two magnetic due to Fe^3+^ and one nonmagnetic due to Y^3+^^18^. There are 40 Fe^3+^ cations in a unit cell, where 16 Fe^3+^ occupy the octahedral (Oct.) sites and 24 Fe^3+^ are evenly distributed over the tetrahedral (Tet.) sites^19^. Moreover, 24 Y^3+^ occupy the dodecahedral (Dod.) sites. The ferrimagnetism in YIG results from both its complex crystal structure with its two unique crystallographic Fe^3+^ sites and a large number of possible exchange interaction with the O^2+^. The network of octahedron-tetrahedron Fe has weak magnetic anisotropy, resulting in the low magnetic damping in YIG^20^. Figure 1b shows the XRD *θ*−2*θ* pattern of the YIG/Gd_3_Ga_5_O_12_ (GGG) bilayers. The peak of YIG indicates a [111] growth orientation on the GGG (111) substrates. Due to the similar crystal structures and close lattice constants between GGG substrate and YIG film, the lattice mismatch between GGG (a = 12.383 Å) and YIG (a = 12.376 Å) is calculated as only 0.057%^20^. The YIG layer can be nicely epitaxially grown on the GGG surface according to the cube-on-cube relationship^21^. According to the 2*d*sin*θ* = n*λ*, the interplane spacing *d*_*111*_ of YIG is obtained as 1.801 Å, which is larger than the value (1.786 Å) in the standard PDF card (No. 98-001-1579). Anomalously large film *d*_111_ interplane spacing clearly indicates rhombohedral distortion of the YIG crystalline lattice (*a*_film_ = *b*_film_ = *c*_film_ and rhombohedral angle *α* < 90°)^22^. The surface topography of GGG substrate and YIG film is obtained by the atomic force microscopy (AFM), which shown in Fig. 1c. The root-mean-square surface (RMS) roughness is 0.08 and 0.27 nm, respectively. It indicates that the pretreated substrate has an atomically stepped GGG surface and facilitates the growth of atomically flat YIG film. Figure 1d shows the normalized static magnetic characteristics for the typical YIG (180 nm) film with the magnetic field applied perpendicular and parallel to the film plane at 300 K. The in-plane magnetic hysteresis loop is relatively square with a coercive field of about 5 Oe. The slanted out-of-plane magnetic hysteresis loop exhibits a coercive field of about 50 Oe and a saturation magnetic field of around 2 kOe. These properties are consistent with previous works and indicate the excellent quality of our epitaxial YIG film^2,21^.

### Measurement of Light-induced spin currents

**Fig. 2:**
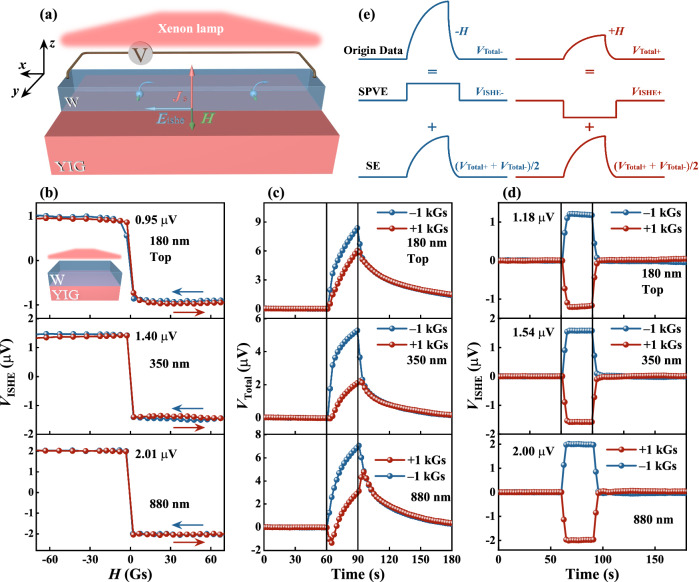
Measurements of the SPVE for different thickness/magnetization configurations by illuminating the top for the W/YIG (180, 350, 880 nm) samples [see the inset of Fig. 2b]. **a** Schematic of the spin photovoltage measurement as Xenon lamp is introduced from the top of the sample, the GGG substrate is not shown here for simplification. **b** shows the magnetic field dependence of *V*_ISHE_ detected. **c** The voltage *V*_Total_ as a function of time was measured at different magnetic field directions along the *y* axis, in response to light that is turned on at 60 s and then turned off at 90 s. **d** The time dependence of *V*_ISHE_ was extracted in Fig. 2c by subtracting thermoelectric signal. **e** Schematic diagram of data processing of SPVE. SE is the abbreviation of the Seebeck Effect, which is a magnetic-field independent thermoelectric signal. The blue and red lines represent the signals at - *H* and + *H*, respectively.

Figure 2a shows a schematic illustration of the sample structure and experimental configuration to measure light-induced spin current. A Xenon lamp was used to illuminate the W/YIG structure from either the top (as shown in Fig. 2a) or the bottom. The magnetic field *H* = 1 kOe is applied along the *y* axis to magnetize the YIG film to saturation, and the *V*_ISHE_ is measured along the *x* axis in the top W layer. The detailed experimental setup for light-induced spin current measurement can be seen Fig. S1 in Supplementary Information. The spin transport process in the W/YIG is briefly as follows: The spin-wave spin current is generated by the light in the YIG via the SPVE. At the W/YIG interface, a spin accumulates and the precessional spin angular momentum in YIG is transferred to the W layer, generating a conduction-electron spin current (*J*_S_). Finally, *J*_S_ in the top W is detected by the voltage via the inverse spin Hall effect (ISHE). The more details will be discussed in theoretical analysis section.

As an electrical method, the ISHE produces an electrical voltage from a spin current via spin-orbit coupling, which has been widely used for detecting spin currents^9,14,23^. We measured the optically generated *V*_ISHE_ for different illumination/magnetic field configurations in W/YIG with the thicknesses for W fixed at 5 nm and for YIG (180, 350, 880 nm), where all samples are of the same dimension and distance between electrical contacts. Figure 2b shows the detected field-dependent *V*_ISHE_ in response to light illuminating the W side of the structure (top-light configuration). The sign of *V*_ISHE_ are reversed by reversing the direction of the magnetic field, confirming the magnetic nature of the SPVE. Figure 2c shows the time dependence of the *V*_Total_ for different directions of magnetic fields in response to light that is first turned on at 60 s and then turned off at 90 s. Under light illumination, the temperature of sample increases due to thermal radiation, leading to an increase in the voltage at both ends of the W electrode, which mainly originates from the thermoelectric effect^1,24^. For experiments on the origin of this thermoelectric signal, please see the section D in the Supplementary Information. The magnitude of the measured voltage varies with the reversal of the direction of the magnetic field, indicating that the signal contains the field-independent voltage produced by the thermoelectric effect and the field-dependent voltage produced by the SPVE.

To minimize the effect of the thermal signals by the temperature gradient, before measuring the variation of *V*_ISHE_ with the magnetic field (Fig. 2b), first wait for 5 min under illumination. Additionally, the thermoelectric signals generated during the collection of *V*_ISHE_ data over time can be removed by processing. Figure 2e illustrates the schematic diagram of data processing for SPVE. The heat signal processing method can be effectively removed by a symmetrization procedure, *V*_ISHE_ can be expressed:

1 $${V}_{{{{{{\rm{ISHE}}}}}}\pm }={V}_{{{{{{\rm{Total}}}}}}\pm }-\left({V}_{{{{{{\rm{Total}}}}}}+}+{V}_{{{{{{\rm{Total}}}}}}-}\right)/2,$$

where *V*_ISHE±_ stands for SPVE signal, *V*_Total_ represents the original data measured by a nanovoltmeter, and the ± symbol represents the direction of the magnetic field. The processing method can effectively subtract the thermoelectric signal (i.e., SE in Fig. 2e and its value equals (*V*_Total+_ + *V*_Total-_)/2. Figure 2d depicts the time dependence of *V*_ISHE_ subtracted from the thermoelectric signal for W (5 nm)/YIG (180, 350, 880 nm) heterojunctions. The *V*_ISHE_ quickly reaches the maximum at 60 s when the light is turned on, and the *V*_ISHE_ detected on YIG with different thicknesses (1.18, 1.54, and 2.00 μV) are close to the saturated values of 0.95, 1.40, and 2.01 μV obtained from the field-sweeping measurements, respectively.

**Fig. 3:**
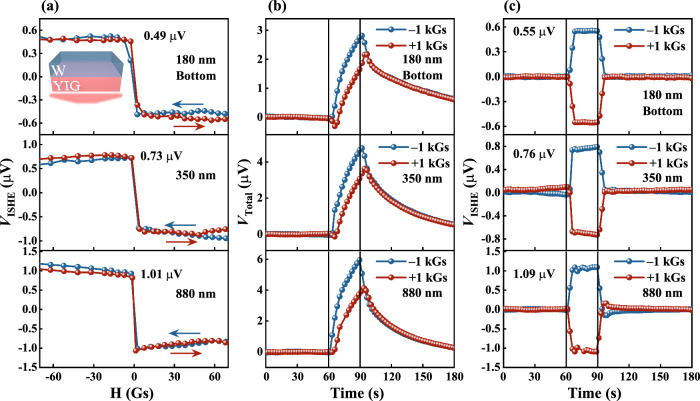
Measurements of the SPVE for different thickness/magnetization configurations by illuminating the bottom for the W/YIG (180, 350, 880 nm) samples [see the inset of Fig. 3a]. **a** The magnetic field dependence of *V*_ISHE_. **b** The voltage *V*_Total_ as a function of time were measured at different magnetic fields directions along the *y* axis, in response to light that was turned on at 60 s and then turned off at 90 s. **c** The time dependence of *V*_ISHE_ were extracted in Fig. 3b by subtracting the thermoelectric signal.

Figure 3 shows the optically generated *V*_ISHE_ in response to illuminating the YIG side for the same sample as described in Fig. 2. The magnetic field dependence of *V*_ISHE_ is displayed in Fig. 3a. The *V*_ISHE_ saturation value of bottom illumination (*V*^bs^_ISHE_) is smaller than that of top illumination (*V*^ts^_ISHE_) for different thicknesses of YIG, and the ratio *V*^bs^_ISHE_/*V*^ts^_ISHE_ is about 0.51 ± 0.04. This is because the light-receiving area of the bottom-light configuration required to fix the sample is about 0.6 times that of the top-light configuration. In addition, a portion of the light energy is absorbed by the GGG substrate, causing a further attenuation of the energy density transmitted to the YIG/W interface. Figure 3b shows the dependence of *V*_Total_ on time at different magnetic field directions. The data are presented in the same behavior as in Fig. 2c. The time dependence of *V*_ISHE_ with the subtracted thermoelectric signal is shown in Fig. 3c. When the magnitude and direction of the magnetic field are fixed, the reversal of the direction of light illumination does not change the sign of the *V*_ISHE_. Conversely, the SSE leads to a change in the sign of *V*_ISHE_ due to the reversal of the direction of the temperature gradient by changing the direction of light illumination. This indicates that the *V*_ISHE_ signal originates from the SPVE rather than the SSE.

### Heat transfer simulation

**Fig. 4:**
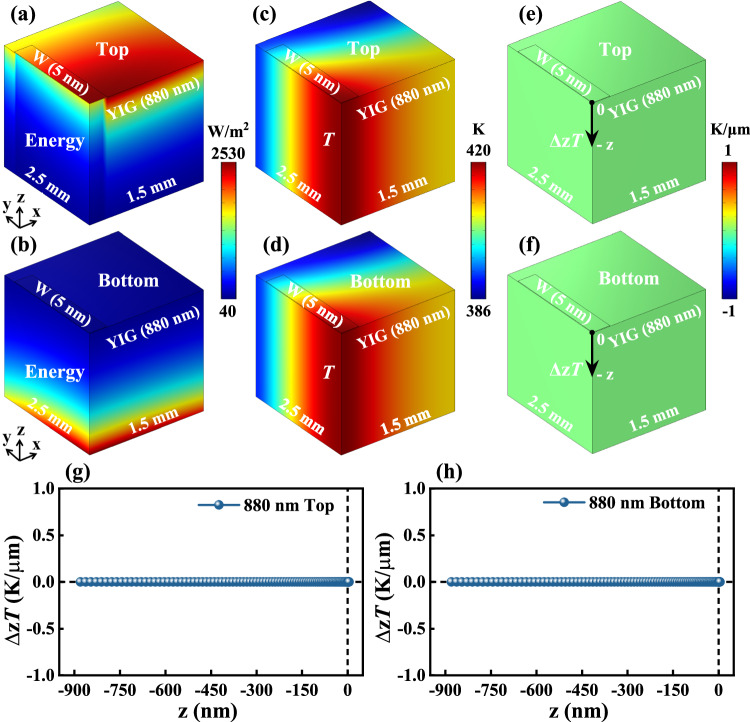
Heat transfer simulation for W (5 nm)/YIG (880 nm) model. **a**, **c,** and **e** are the three-dimensional color maps of the energy density distribution, temperature distribution and the Δ_z_*T*, for the top-light configuration with a large-spot size, respectively. **b**, **d,** and **f** show the corresponding bottom-light simulation results. The Δ_z_*T* are plotted as a function of distance from the interface for (**g**) top-light and (**h**) bottom-light configurations, respectively.

To further eliminate possible SSE in this experiment, the temperature gradient (Δ_z_*T*) along z direction will be provided through heat transfer simulation. For the case of the shorter light penetration depth and the thicker sample, it is easier to generate light-induced temperature gradient^13,14^. Hence, the larger-spot light with short-wavelength (450 nm), which is the case of low light intensity, is used for heat transfer simulation on a W (5 nm)/YIG (880 nm) model. The simulation results are shown in Fig. 4. Figure 4a, b show the distribution of energy density when light is shed on the top and bottom of the W (5 nm)/YIG (880 nm) model, respectively. The results verify that the thicker YIG (880 nm) cannot be penetrated by the short-wavelength light and the energy will be distributed on the surface, when the top or bottom of model is irradiated by a large-spot light. However, there is no obvious difference in the temperature distribution under different directions of light illumination (see Fig. 4c, d). Hence, the Δ_z_*T* is uniform, for the top-light and bottom-light configuration (as shown in Fig. 4e, f). Figure 4g, h show the change of the Δ_z_*T* with the distance from the interface for top-light and bottom-light configuration, respectively. Here, the Δ_z_*T* is only 10^−4 ^K/μm, and such a small magnitude order of Δ_z_*T* is insufficient to induce the significant SSE.

In our experiment, the large-spot Xenon lamp with low energy (i.e., low light intensity) is used, so the Δ_z_*T* inside the sample will be very small. After reversing the direction of illumination, no reversal *V*_ISHE_ is observed in different thicknesses of YIG samples. Thus, the light-induced spin current we observed in the experiment belongs to the photo-excited spin current (i.e., SPVE). This is consistent with these previous photo-excited spin current reports where a large-spot light is also employed^1,10,12^.

Note that light-induced spin current is also considered as a light-thermally excited spin current (i.e., SSE), and the smaller-spot laser with higher energy (i.e., high light intensity) is used, and the thickness of samples is generally in the range of micrometer^13,14,25^. Consequently, a large Δ_z_*T* and reversal of the Δ_z_*T* sign with a reversed illumination direction led to the flipped sign of *V*_ISHE_. Similarly, if a high light intensity is used to illuminate our sample, the SSE rather than SPVE should be observed. To validate this hypothesis, we choose the 405 nm laser converged by a convex lens, which is a smaller-spot laser with higher energy, and utilize it to illuminate the W (5 nm)/YIG (880 nm) sample. As we anticipated, the reversal of illumination direction causes a reversal sign of the *V*_ISHE_. This experimental observation confirms that the spin current observed under this condition is mainly related to SSE [the detailed results shown in Fig. S3a, b]. Moreover, the corresponding simulation for the smaller-spot laser with higher energy is also conducted, and the simulated results are shown in Fig. S3c–f. This simulated results also show that after reversing the illumination direction, the magnitude of Δ_z_*T* can reach up to 0.94 K/μm (≫10^−4 ^K/μm for large-spot light with low-energy) and the sign of Δ_z_*T* also is reversed, further proving the above analysis.

Therefore, Both SPVE and SSE contribute to the generation of spin current under light illumination. However, SPVE dominates under low light intensity, whereas SSE plays the dominant role under high light intensity. The debate on SPVE and SSE in YIG system is resolved.

### Theoretical analysis

Next, we will discuss the SPVE mechanism in YIG system. Currently, a shift current model is proposed to explain the physical mechanism of photo-excited spin currents. A brief introduction to this model can be found in the Supplementary Information. According to the shift current model, the shift vector is odd under ***P*** symmetry, that is, ${R}_{{nm}}^{a}\left(k\right)=-{R}_{{nm}}^{a}\left(-k\right)$. Therefore, a material with ***P*** symmetry, the integration in Eq. (S3) is zero, and thereby the shift current conductivity is equal to zero (${\sigma }_{{bb}}^{a}=0$)^3,15^. The shift current model restricts the spin photovoltaic response to 20 out of the 21 non-centrosymmetric point groups^16^. The schematic for the symmetry operations and stereographic projection of point group *m3m* (*O*_*h*_) of YIG is given in Fig. S6a, b [see Supplementary Information], respectively. The point group *m3m* (*O*_*h*_) is a centrosymmetric point group with 48 symmetry operations. In bulk state, the lattice constant of YIG (12.381 Å) is smaller than that of the GGG substrate (12.385 Å)^22^. From the XRD spectrum of the epitaxial YIG film on GGG substrate (see Fig. 1b), it can be observed that the YIG film is subjected to in-plane compressive stress, resulting in an increase in the *d*_111_ of the YIG (111) plane from 1.787 Å to 1.797 Å. When YIG deforms along the [111] direction, its point group changes to centrosymmetric *−3m* (*D*_*3d*_). The schematic for the symmetry operations and stereographic projection of point group -*3m* are given in Supplementary Information Fig. S6c, d, respectively. According to symmetry requirements, no third-rank tensor elements is satisfied with the two point groups.

**Fig. 5:**
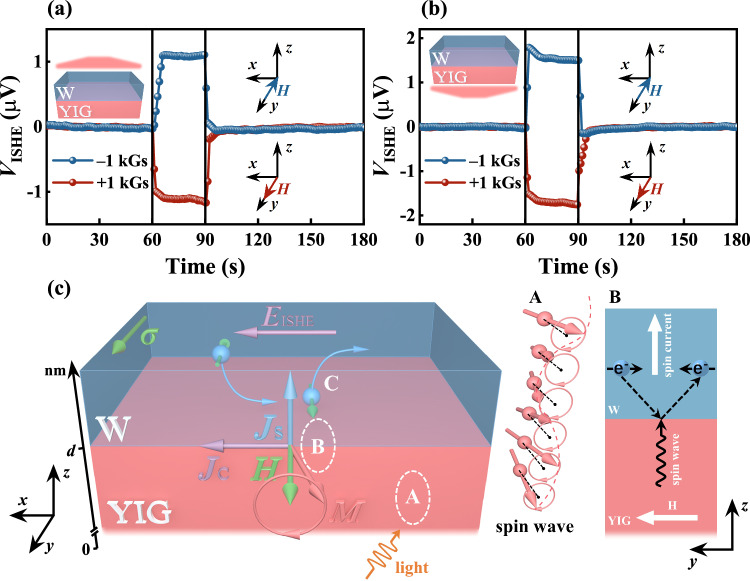
The physical mechanism of SPVE. *V*_ISHE_ measured via a red laser with a wavelength of 808 nm. The temporal evolution of *V*_ISHE_ signals for different directions of magnetic fields in response to a laser turned on at 60 s and off at 90 s with (**a**) top-light and (**b**) bottom-light configurations, respectively. **c** Sketch of the physical mechanism on SPVE in W/YIG system.

On the other hand, the shift current model suggests that a shift of the wave packet is generated in real space when an electron is pumped from the valence band to the conduction band by linearly polarized light. YIG films have a bandgap of approximately 2.7 eV, and thus, only the light with a wavelength shorter than 456 nm can induce excitations in YIG films^26^. We employed a red laser with a wavelength of 808 nm (25 mW/cm^2^) to measure the time-dependent variation of *V*_ISHE_ under different magnetic field directions when illuminating the top (Fig. 5a) and bottom (Fig. 5b) of W (5 nm)/YIG (880 nm). The result demonstrates the red laser illumination also gives rise to a notable spin current signal. Hence, the shift current model is not valid for the SPVE observed in W/YIG system.

In general, a spin current refers to the flow of spin angular momentum. In solids, there are two types of carriers for non-equilibrium spin currents^26^. In magnetic materials, one is the conduction-electron spin current, which depends on the exchange of angular momentum of itinerant electrons. Another is the spin-wave spin current which depends on the transfer of spin angular momentum. Without free electrons in the Fe^3+^ and Y^3+^ ions, YIG is an excellent insulating material. Additionally, because of a very low spin damping, it is commonly considered to exhibit spin-wave spin current^13,18,26^. As shown in Figs. 2 and 3, the SPVE significantly enhances with an increase in the thickness of the YIG film, regardless of illumination direction. These results indicate that the photo-excited spin current is not only generated at the W/YIG interface. As shown in Fig. 5c, the propagation process of the spin current excited by photons in W/YIG is divided into three parts: within the YIG layer (A), at the W/YIG interface (B), and within the W layer (C).

Firstly, the process of A shows the propagation of the spin waves in YIG. The study of the ground state of YIG can only provide insights into the static properties of the system, i.e., properties measured at 0 K. A magnetic field is applied along the *y* direction of YIG, thus the magnetic moments from uncompensated spins of the Tet. Fe^3+^ ions are aligned along the *y* direction. When light is applied in this scenario, an individual spin is perturbed by photon, deviating from its equilibrium state. Due to the interactions between this spin and its neighboring spins, the perturbation induced by photon propagates throughout the entire system and forms the spin waves in YIG.

Secondly, there is a conversion from spin waves to spin current in the process of B. The spin angular momentum of the precession in YIG is transferred to the W layer by the s-d interaction, which conserves total angular momentum^17,27,28^. The boundary condition at the W/YIG interface can be expressed as^17^:

2 $${j}_{m}\left({d}^{-}\right)=\left(-{\mu }_{{{{{{\rm{B}}}}}}}/e\right)\left[ \, {j}_{s}^{\uparrow }\left({d}^{+}\right)-{j}_{s}^{\downarrow }\left({d}^{+}\right)\right],$$

Where e is electron charge, *μ*_B_ is the Bohr magneton, *j*_m_ and *j*_s_ denote the magnon current density of YIG and spin current density of W, respectively. The interface YIG/GGG is assigned at z = 0, and *d* corresponds to the thickness of YIG. The symbol - (+) represents the decrease (increase) in spin angular momentum on the YIG (W) side at the interface, and ↑ (↓) corresponds to the spin-up (down) component.

The physics of the Eq. (2) is fairly transparent: the left term describes the flow of magnons generated under the action of photons, and the right term describes the spin current generated by the accumulation of magnons. Thus, the magnon current at the interface will be fully converted into the conduction-electron spin current in the W layer. In fact, spin-flop scattering caused by impurities and defects present at the W/YIG interface will reduce the outgoing spin angular momentum^4^.

Finally, the spin angular momentum is transferred from spin precession to conduction-electron spins, forming a pure spin current *J*_S_ in the W layer (the process of C). This spin current in the W is detected by the voltage via the ISHE. The process that converts *J*_S_ into a charge current *J*_C_ is described. Assuming the spin-polarization vector direction as *σ*, two electrons moving in the opposite direction of *J*_S_ have opposite spins: one parallel to *σ* and the other anti-parallel to *σ* in the pure spin current in W layer. These two electrons are bent to the same direction and induce *J*c perpendicular to *J*s via spin-orbit coupling. The relationship between *J*s and *J*c is thus given by the following equation^9,24,29^:

3 $${J}_{{{{{{\rm{C}}}}}}}={\theta }_{{{{{{\rm{SH}}}}}}}\frac{2e}{{{\hslash }}}{J}_{{{{{{\rm{S}}}}}}}\times \sigma,$$

where *θ*_SH_ represents the spin Hall angle (which is used to characterize the efficiency of the ISHE, and *θ*_SH_ of W is negative) and ℏ is in a material reduced Planck’s constant.

*V*_ISHE_ obtained from the data detected by the nanovoltmeter is given by^30^:

4 $${V}_{{{{{{\rm{ISHE}}}}}}}=\frac{A}{{\sigma }_{{{{{{\rm{C}}}}}}}}{J}_{C}=\frac{A}{{\sigma }_{{{{{{\rm{C}}}}}}}}{\theta }_{{{{{{\rm{SH}}}}}}}\frac{2e}{{{\hslash }}}{J}_{{{{{{\rm{S}}}}}}}\times \sigma,$$

where *A* is a coefficient related to the thickness of the film and *σ*_C_ is the electrical conductivity.

## Discussion

In summary, we have prepared W/YIG (111) bilayers heterostructure by PLD method. SPVE and optically induced SSE are experimentally observed in this heterostructure via different light sources. Combined with heat transfer simulation in W/YIG model, it is found that large-spot light with low-energy produces photo-excited spin currents (i.e., SPVE), whereas small-spot light with high-energy generates light-thermally excited spin currents (i.e., SSE). Under large-spot light with low-energy illumination, SPVE is predominant and SSE is omitted thanks to the negligible temperature gradient. By means of symmetry analysis and experimental results, the model of shift current for SPVE is excluded. Based on the conservation of total angular momentum and the ISHE, a more reasonable model for SPVE in this system is presented. Our finding deepens the understanding for the origin of light-induced spin current in heavy metal/magnetic insulator bilayers, and SPVE with ultrafast time response may enable the design for high-speed spin photovoltaic devices.

## Methods

### Sample preparation

The high-quality epitaxial W/YIG (111) bilayers were fabricated on (111)-oriented GGG substrate (5 × 3 mm^2^). The GGG (111) substrate was ultrasonically cleaned with acetone, isopropanol and deionized water, and then annealed for 6 h in air at 900 °C. Epitaxial YIG films with different thickness were deposited on (111)-oriented GGG substrates by pulsed-laser deposition (PLD) with a KrF excimer laser (*λ* = 248 nm). Under an oxygen pressure of 83 mTorr, films were grown at 750 °C with a laser energy density of 1-2 J/cm^2^ and a repetition rate of 6 Hz. Subsequently, the films were annealed at 375 Torr of oxygen pressure and slowly cooled down. Then the samples were capped by 5-nm-W spin current detectors using a bar-shaped mask (0.4 × 4.4 mm^2^) by magnetron sputtering with Ar pressure at 2 mTorr.

### Sample characterization

The layer thicknesses were calibrated by x-ray reflectivity (XRR) and controlled by the deposition time. The crystal structure of YIG film was characterized by X-ray diffraction (XRD, Bruker D8 Discover, Cu-Kα radiation, *λ* = 1.54056 Å). The surface characteristics of YIG and GGG substrate were detected by the atomic force microscopy (AFM, Cypher, Asylum Research), and the RMS is used to describe the surface roughness. The magnetic hysteresis loops of the YIG thin film at 300 K was recorded by magnetic property measurement system (MPMS3, Quantum Design). The Seebeck coefficients were measured by a Hall-effect measurement system (Linseis, HCS 1) with the Seebeck coefficient characterization.

### SPVE measurements

The SPVE measurements were carried out in a home-made measurement system. The sample was placed in a vacuum chamber to reduce the noise signal in the process of measurement, and a Xenon lamp (~300 mW/cm^2^) or laser was irradiated on the top or bottom of the sample. The magnetic field was provided by an electromagnet, and a nanovoltmeter (Keithley, 2182 A) was used to measure *V*_ISHE_ across the W length. All measurements were carried at room temperature, and the schematic diagram of the experimental setup and sample structure is shown in Fig. S1.

### Heat transfer simulation model

By the finite element method in COMSOL multiphysics modeling, local and non-local heating processes in transparent media with non-uniform temperature gradients is simulated^31^. When the size of the heated object and the size of the laser spot are both much larger than the wavelength, the Beer-Lambert law can be used to simulate the light-induced thermal conduction process in transparent materials well.

According to the energy conservation, the temperature field can be expressed as:

5 $$\rho {C}_{{{{{{\rm{P}}}}}}}\frac{\partial T}{\partial t}-\nabla \cdot \left(k\nabla T\right)={Q}_{{{{{{\rm{r}}}}}}},$$

where *Q*r is heat source term, *ρ* is the mass density, *C*_P_ is the heat capacity at constant pressure, and *k* is the thermal conductivity^32,33^.

The simplified Beer-Lambert Law can be expressed as:

6 $$\frac{{e}_{i}}{{||}{e}_{i}{||}}\cdot \nabla {I}_{i}=-\kappa {I}_{i}$$

where *e*_i_ represents the direction of the light beam and *I* is radiative intensity. When a light beam passes through a medium, energy is absorbed, and the radiative heat source term can be expressed as:

7 $${Q}_{{{{{{\rm{r}}}}}}}={\sum}_{i}\kappa {I}_{i}$$

During the heat transfer processes, temperature *T* affects radiative intensity *I* through the absorption coefficient *κ*, and radiative intensity *I* affects temperature *T* through the radiative heat source *Q*. The more details were discussed in the section B of the Supplementary Information.

### Reporting Summary

Further information on research design is available in the Nature Portfolio Reporting Summary linked to this article.

### Supplementary information

Supplementary Information

Lasing Reporting Summary

## Data Availability

The data that support the findings of this study have been included in the main text and the Supplementary Information. Source data are provided in this paper. 10.6084/m9.figshare.25712169.
