# Supplementary material for: Origin of the light-induced spin currents in heavy metal/magnetic insulator bilayers
Source: Nat Commun. 2024 May 22;15:4362. doi: 10.1038/s41467-024-48710-6 (PMC11111453; doi:10.1038/s41467-024-48710-6)
Supplement: Supplementary file 1 — Supplementary Information [file 41467_2024_48710_MOESM1_ESM.pdf]

Supplementary Information for "Origin of the light-induced spin currents in heavy metal/magnetic insulator bilayers"

Hongru Wang,[1] Jing Meng,[1] Jianjun Lin,[1] Bin Xu,[1] Hai Ma,[1] Yucheng Kan,[1] Rui Chen,[1] Lujun Huang,[2] Ye Chen,[1] Fangyu Yue,[1] Chun-Gang Duan,[1] Junhao Chu,[1, 3] Lin Sun[1,*]

[1]Key Laboratory of Polar Materials and Devices (MOE), Department of Electronics, East China Normal University, Shanghai 200241, China

[2]The Extreme Optoelectromechamics Laboratory (XXL), School of Physics and Electronic Science, East China Normal University, Shanghai 200241, China

[3]Institute of Optoelectronics, Fudan University, Shanghai 200438, China

Corresponding author: lsun@ee.ecnu.edu.cn

A. Spin photovoltaic effect (SPVE) measurements

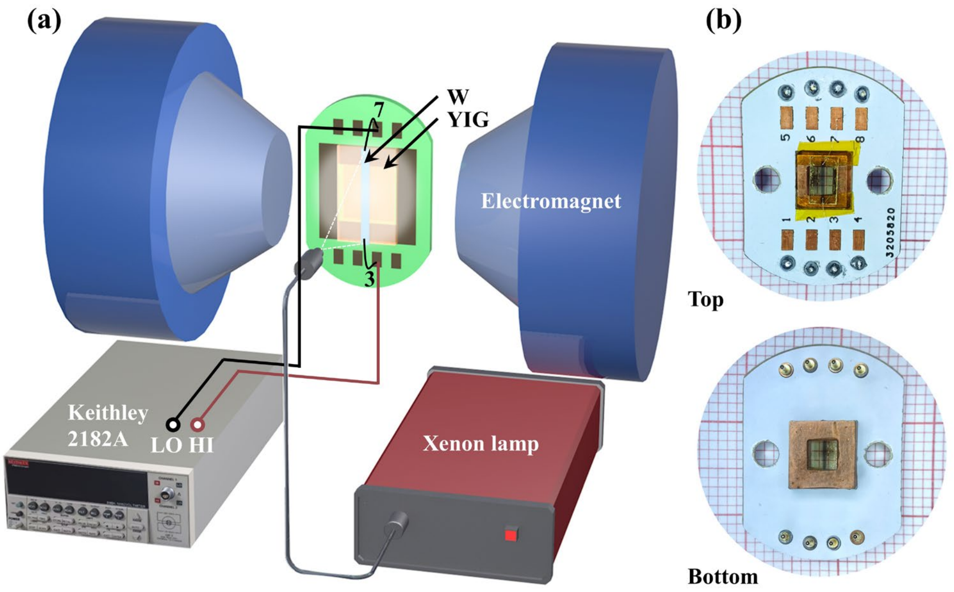

Fig. S1. Experimental set-up. (a) A schematic diagram of the experimental setup for light-induced spin current measurement (HI was connected to the number 3 of electrode, and LO was connected to the number 7). (b) A photograph of the front and back of the sample holder. The metal (Cu) pads at both ends of the W electrode are connected for the purpose of connecting to the nanovoltmeter (Keithley, 2182A) to test V_{ISHE}.

B. Heat transfer simulation model

Table S1 Mass density (ρ), thermal conductivity (κ), heat capacity (C), penetration depth (δ) and interfacial thermal resistance (R) used in the simulation.

	Mass density ρ (kg m^{-3})	Thermal conductivity κ (W m^{-1} K^{-1})	Heat capacity C (J kg^{-1} K^{-1})	Penetration depth $\delta_{450\ \mathrm{nm}}$ (nm)	Interfacial thermal resistance R (K m^2 W^{-1})
Pt	21450[1]	20[2]	130[1]	5.8[3]	-
W	19250	185[4]	132.45[5]	10.51[6]	-
YIG	5170[7]	6.63[7]	570[1, 8, 9]	302[3]	-
Pt/YIG	-	-	-	-	$5.9*10^{-9}$ [10]
W/YIG	-	-	-	-	$5.9*10^{-9}$ [10]

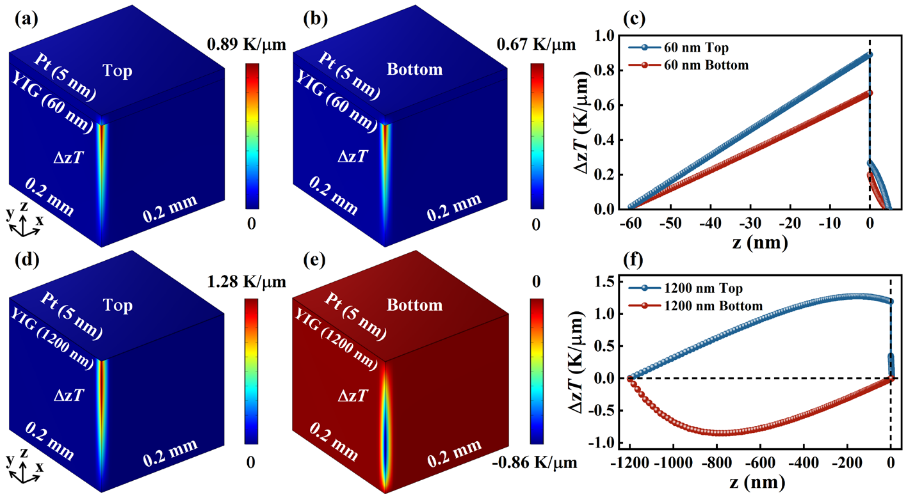

Fig. S2 Heat transfer simulation for W (5 nm)/YIG (60 and 1200 nm) model. Three-dimensional color maps of the $\Delta_z T$ distribution by COMSOL simulation for top-light, bottom-light configurations, and the temperature gradient excited by a local blue laser as a function of distance from the interface for (a)-(c) Pt (5 nm)/YIG (60 nm) system and (d)-(f) the Pt (5 nm)/YIG (1200 nm) model, respectively.

The Pt/$Y_3Fe_5O_{12}$ (YIG) system was chosen to simulate the absorption of laser in a transparent medium. The parameters used in the simulations are indicated in Table S1. At room temperature, the interface conductance in most metals/oxide systems were found range from 50 to 300 MW m^{-2} K^{-1} [10]. For instance, the interface conductance of Pt/YIG is 170 MW m^{-2} K^{-1} (interfacial thermal resistance， $5.9*10^{-9}$ K m^2 W^{-1})[10]. In the simulation, the interfacial thermal resistance was set using $5.9*10^{-9}$ K m^2 W^{-1} for both Pt/YIG and W/YIG. It should be noted that this is an estimated value for W/YIG. The light used in the simulation is a Gaussian beam with an average power of 10 mW and a standard deviation of 10 micron (small-spot light with high-energy)[3, 11]. In light-induced thermal excitation spin current reports, it has been observed that reversing the direction of illumination from top to bottom leads to a reversal in the inverse spin Hall voltage (V_{ISHE}), where the light sources used in most cases are low-wavelength monochromatic light. Herein, we simulate the effect using blue light with low penetration depth[3, 11]. To highlight the temperature distribution near the laser spot, the dimensions within the model surface were set to 0.2 mm × 0.2 mm, and the view was scaled to a cube.

Fig. S2a and S2b show three-dimensional color maps of the temperature gradient ($\Delta_z T$) along z direction when light is added to the top and bottom of the Pt (5 nm)/YIG (60 nm) sample, respectively. Fig. S2c shows the dependence of $\Delta_z T$ on the distance from the interface. The $\Delta_z T$ is always positive when light is added to the top and bottom of the Pt (5 nm)/YIG (60 nm) sample. For the 1200 nm-YIG film, as shown in Fig. S2d-S2f, the simulated results show $\Delta_z T$ is positive when light is added to the top of sample.

While the light is reversed, a negative $\Delta_z T$ is generated in YIG due to the weak penetration depth of blue light. As shown in Fig. S2c and S2f, a discontinuous change in $\Delta_z T$ occurs at the interface of Pt (5 nm)/YIG. By analysis, YIG and Pt (5 nm), as a semi-transparent media, allow partial light penetration through the Pt layer to the YIG layer. Therefore, in this process, a discontinuous variation in simulated $\Delta_z T$ results from different thermal parameters (thermal conductivity, heat capacity, etc.) of Pt and YIG. This result is consistent with previous experimental and simulation reports, indicating the feasibility of using this model for simulation[3, 11, 12]. The simulations in this paper are based on this model.

In the simulation, the $\Delta_z T$ on Pt/YIG can be divided into the interface temperature gradient ($\Delta_z T_{\mathrm{Int}}$) caused by the action of the Pt heater and the bulk temperature gradient ($\Delta_z T_{\mathrm{Bulk}}$) caused by the distribution of light energy in YIG[3]. For the 60 nm-YIG film, the shorter penetration depth of Pt results in higher energy on Pt under top-light illumination configuration. For the bottom-light configuration, there is still a large energy density on Pt since the YIG (60 nm) of the translucent medium is relatively thin. Therefore, Pt acts as a heater in the Pt (5 nm)/YIG (60 nm) model, and $\Delta_z T$ caused by this phenomenon is defined as the $\Delta_z T_{\mathrm{Int}}$. In this model, the $\Delta_z T_{\mathrm{Int}}$ is dominant and $\Delta_z T_{\mathrm{Bullk}}$ has little contributes to the $\Delta_z T$, which results in a fixed direction of the $\Delta_z T$ regardless of the direction of light (see Fig. S2c). It is noted that $\Delta_z T_{\mathrm{Bulk}}$ dominates in the $\Delta_z T$ for the thicker YIG (1200 nm). In the Pt (5 nm)/YIG (1200 nm) model, as the direction of light illumination changes, the direction of $\Delta_z T_{\mathrm{Bulk}}$ reverses, leading to the reversal of $\Delta_z T$ (see Fig. S2f).

C. Heat transfer simulation of W/YIG (880 nm) under small-spot irradiation

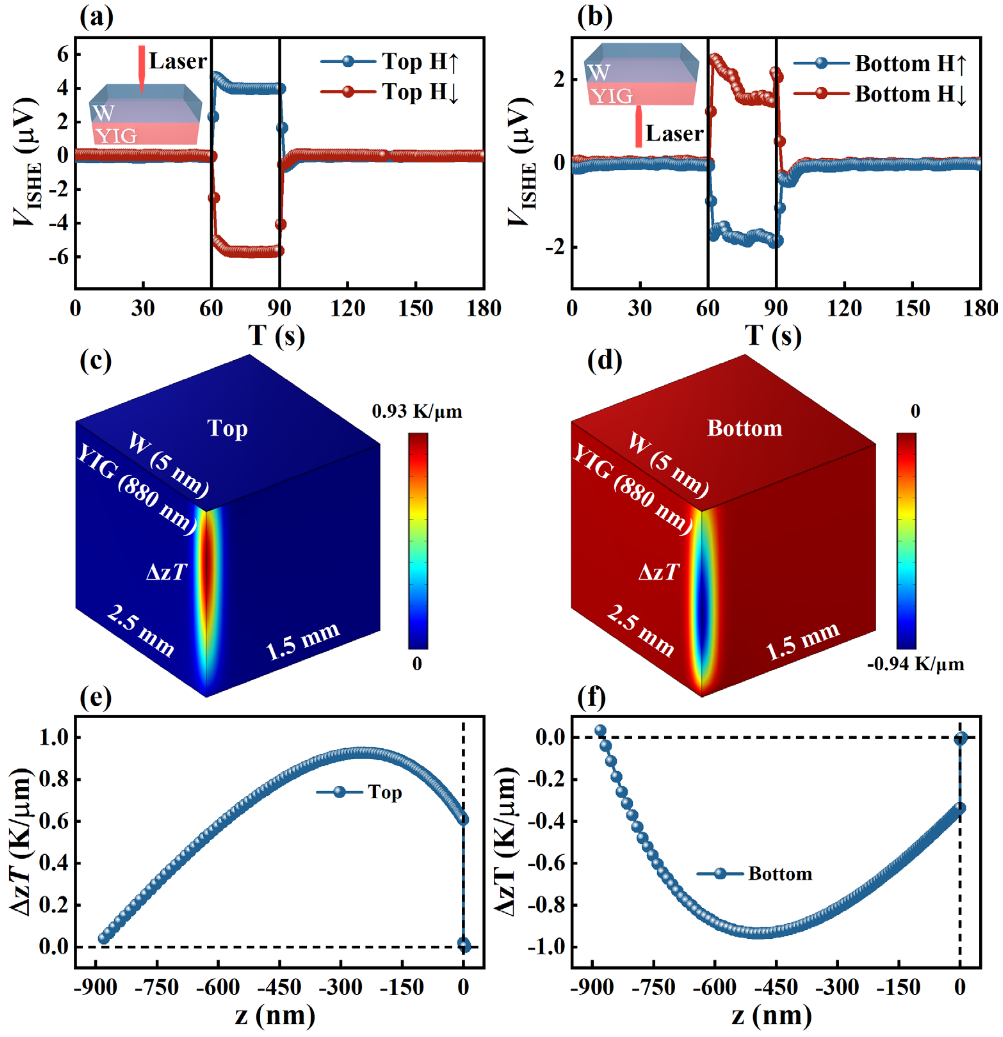

Fig. S3 V_{ISHE} measured via using laser as the light source and heat transfer simulation. The temporal evolution of V_{ISHE} signals for different directions of magnetic fields in response to a laser turned on at 60 s and off at 90 s with (a) top-light and (b) bottom-light configurations, respectively. The three-dimensional color maps of $\Delta_z T$ distribution by COMSOL simulation for (c) top-light and (d) bottom-light configurations for W (5 nm)/YIG (880 nm). (e) and (f) are corresponding the temperature gradient excited by a local blue laser as a function of distance from the interface.

D. The origin of thermoelectric signals

The thermoelectric effect is attributed to the small difference in temperature at both ends of the sample. Similar phenomena that the temperatures respond to the "on" and "off" operations of the light in a manner much slower than that of the voltage have been observed by Ellsworth et al[13]. The voltage signal changes with time in the exactly same manner as the temperature difference between the two electrodes for the voltage measurements. Considering the small magnitude oder of the test voltage (10^{-6} V), even minor temperature differences in the actual testing environment could generate thermoelectric signals. To verify this result, first demagnetize the sample along the *y*-axis first and set the magnetic field to zero, and the SPVE does not work and make no contribution to voltage signal. The related experiments are designed by changing the position of light source $\Delta x \approx 3$ mm along *x* directions as illustrated in Fig. S4. That is, the light spot is located on the upper position [see Fig. S4a], the middle position [see Fig. S4c] and the lower position [see Fig. S4e], respectively. And the corresponding the time dependence of the V_{ISHE} of W/YIG were measured in Fig. S4b, S4d, and S4f. At t = 60 s, a voltage gradually reaches its maximum value as the sample was illuminated. After turning off the illumination at t = 300 s, voltage gradually decreases to zero at approximately t = 400 s. We attribute the gradual change in voltage to thermal relaxation, with the corresponding voltage primarily being a thermoelectric signal. Experimental results indicate that as the illumination position moves from top to bottom, the saturation value of the voltage decreases. When the illumination is close to the lower electrode, the voltage polarity reverses, indicating a reversal of the in-plane temperature

difference during the movement process.

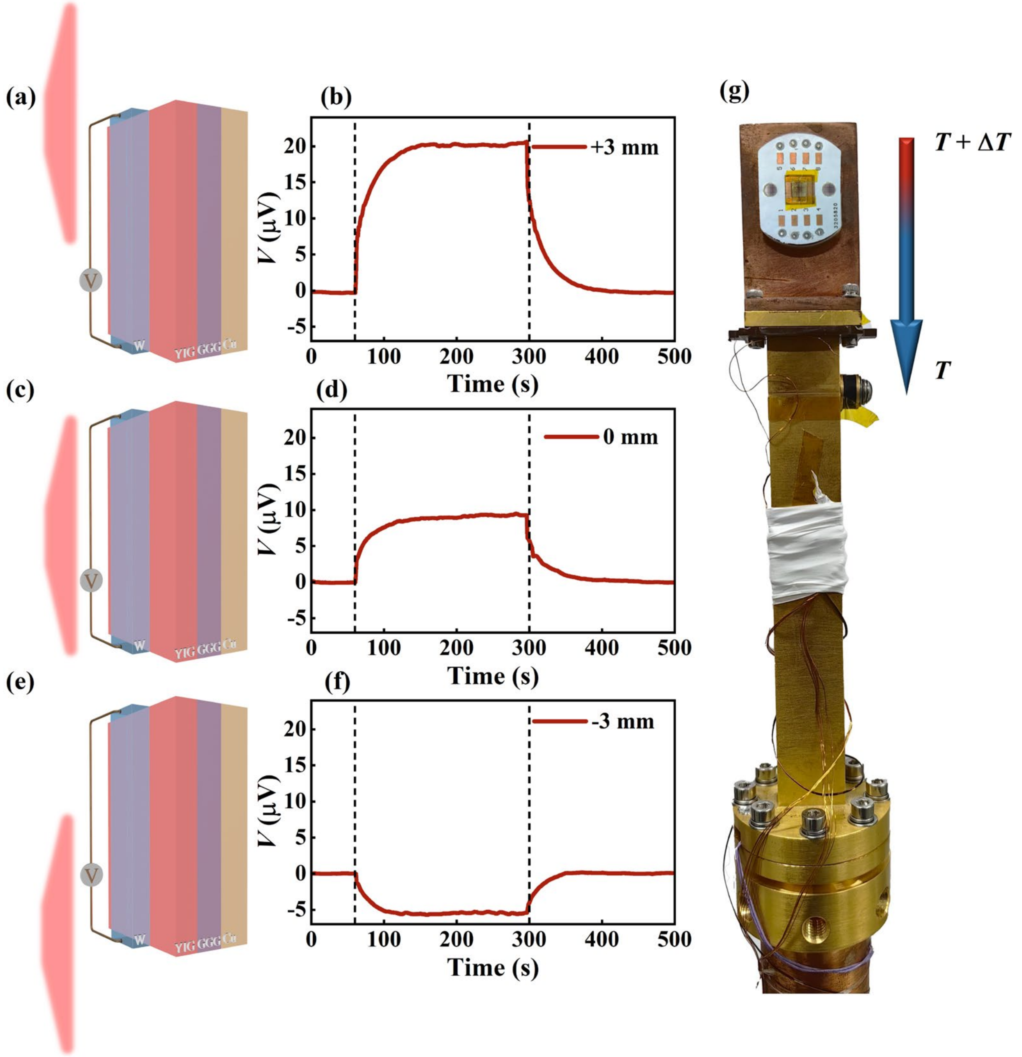

Fig. S4 Thermoelectric signals of W/YIG sample under light. Schematic diagram of the light spot located on the front side of the W/YIG sample (a) at the upper end, (c) at the equilibrium position, and (e) at the lower end; The V-Time curve of the light spot located at (b) the upper position, (d) equilibrium position, and (f) the lower position of the front side of the W/YIG sample. (g) presents a physical illustration of the sample holder.

It is intriguing that there is also a thermoelectric signal when the illumination is directly above the sample. By analysis, the shape of the sample holder prevents uniform

heat dissipation at both ends of the sample during measurement, resulting in a slight temperature difference that generates a thermoelectric signal. Fig. S4g presents a physical illustration of the sample holder, where the Cu holder in contact with the sample facilitates more efficient heat dissipation at the lower end, causing a slightly higher temperature at the upper end of the sample.

To further confirm that the thermoelectric signal originates from the in-plane temperature gradient on the W (5nm)/YIG sample, the Seebeck coefficient (*S*) was measured. The *S* is calculated as $S = \Delta V/\Delta T$, where ΔV is the voltage and ΔT is the change temperature. Therefore, the ΔV-ΔT curve should be a straight line passing through zero. In practice, due to the offset voltage in the measuring apparatus, a voltage drop takes place at a zero-temperature difference. To eliminate the errors as much as possible, it is more accurate for the slope method to measure *S* than single-point measurement[14].

To obtain the accurate value of *S*, we first recalibrated the experimental set-up parameter by measuring the *S* of a standard constantan sample (this sample is only for calibration), and then measured the *S* of W (5 nm)/YIG again. The photograph of sample holder is shown in Fig. S5a. Fig. S5b shows the Voltage vs Temperature difference of the sample of W (5nm)/YIG after calibrating the testing system. After obtaining the raw data, only relative Seebeck coefficient (S_{rel}) can be evaluated because the probes also produce a voltage[14]. In Fig. S5b, the blue symbol represents the raw data, and the slope obtained through linear fitting corresponds to the slope S_{rel}, with a value of 17.45 μV/K. The slope $S_{rel} = S_{pro} + S_{abs}$, where S_{pro} represents the *S* of probe.

The S_{abs} is obtained by subtracting the calibrated S_{pro} (15.74 μV/K) from the S_{rel}, resulting in a value of 1.71 μV/K. It is 6.99 μV/K when converted to that of relative to Pt ($S_{rel,\ Pt}$), which is consistent with previous literature (S_{abs}, 1.00 μV/K; $S_{rel,\ Pt}$, 6.28 μV/K)[15]**.** Due to YIG (an oxide insulator) with the negligible carrier concentration, the Seebeck effect of single YIG films is extremely small. Therefore, the contribution of the Seebeck voltage caused by the in-plane temperature difference originates from the 5 nm-W film.

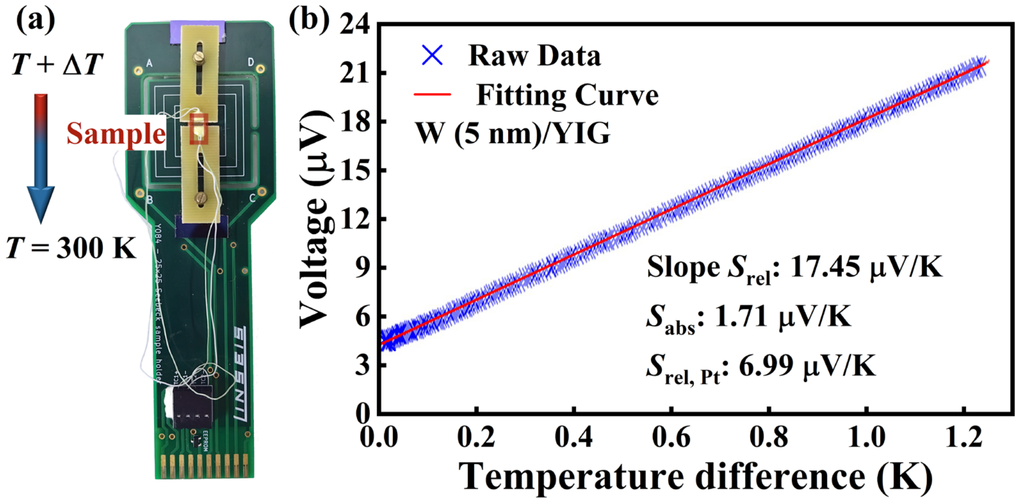

Fig. S5 The *S* of W/YIG sample. (a) Photograph of sample holder for measuring the *S*. (b) The voltage-temperature difference curve of the W (5 nm)/YIG sample.

E. Introduction to the shift current model

The SPVE can generate spin currents in a non-contact and ultrafast manner through optical excitation. The antiferromagnetic structure has two sublattices with opposite spin polarization, and the spin bulk photovoltaic effect is observed when the inversion symmetry (***P***) of these two sublattices is broken[15, 16]. In this scenario, the photogenerated currents in two sets of opposite sublattices carry different types of spins and propagate in opposite directions. This results in the cancellation of the charge current ($j_{\mathrm{c}} = j_{\uparrow} + j_{\downarrow} = 0$), but the two kind of spin currents in different directions combine to form a pure spin current ($j_{\mathrm{s}} = \frac{\hbar}{2e}(j_{\uparrow} - j_{\downarrow}) \neq 0$)[17].

Through this model, the photoelectric effect can be separately analyzed in ferrimagnetic with two spin-up and spin-down sublattices. When an electron is pumped from the valence band to the conduction band by linearly polarized light, it generates a shift of wave packet in real space[16, 17]. First, considering the nonlinear light, the shift current can be described as:

$$j_{\mathrm{shift}}^{a} = 2\zeta_{bc}^{a}(0; \omega, -\omega)E^{b}(\omega)E^{c}(-\omega). \qquad \text{(S1)}$$

For linearly polarized light b = c, Eq. (S1) can be further simplified as

$$j_{\mathrm{shift}}^{a} = 2\sigma_{bb}^{a}(0; \omega, -\omega)E^{b}(\omega)E^{b}(-\omega), \qquad \text{(S2)}$$

where *a*, *b* and *c* are the Cartesian index. *a* indicates the direction of the current, *ω* is the frequency of light, $E^{b}(\omega)$ represents the Fourier component along the *b* direction of the electric field at *ω*[15, 16, 18].

The shift current conductivity is:

$$\sigma_{bb}^{a}(0; \omega, -\omega) = -\frac{\pi e^{3}}{2\hbar^{2}} \int_{BZ} \frac{d^{3}k}{(2\pi)^{3}} \sum_{n,m} f_{nm} R_{nm}^{a} |r_{nm}^{b}|^{2} \delta(\omega_{mn} - \omega), \qquad \text{(S3)}$$

where $f_{nm} = f_n - f_m$ is the difference of occupation factors between bands n and m, $r_{nm} = \langle m|r|n\rangle$ is a positional operator and $\hbar\omega_{mn} = \varepsilon_m - \varepsilon_n$ is the difference of band energies[15, 16, 18]. The shift vector R_{nm}^a can be defined as:

$$R_{nm}^a = \frac{\partial \Phi_{nm}}{\partial k^a} + \xi_{nn}^a - \xi_{mm}^a, \tag{S4}$$

where Φ_{nm}^a is the phase factor of the interband Berry connection, ξ_{nn}^a is the intraband Berry connection matrix along a direction[15, 16, 18]. Considering spin polarization, the shift current j_{shift}^a should be calculated separately for the spin-up and spin-down sublattices:

$$j_{\text{shift}}^{a\,S} = j_{\text{shift}}^{a\,\uparrow} - j_{\text{shift}}^{a\,\downarrow}. \tag{S5}$$

The shift vector is odd function under ***P*** symmetry, that is, $R_{nm}^a(k) = -R_{nm}^a(-k)$. Therefore, the integration in Eq. (S3) is enforced to be zero in a ***P*** symmetric material, leading to $j_{\text{shift}}^{a\,S} = 0$[16]. The model is suitable for SPVE in crystals with broken ***P*** symmetry.

F. Symmetry analysis of YIG

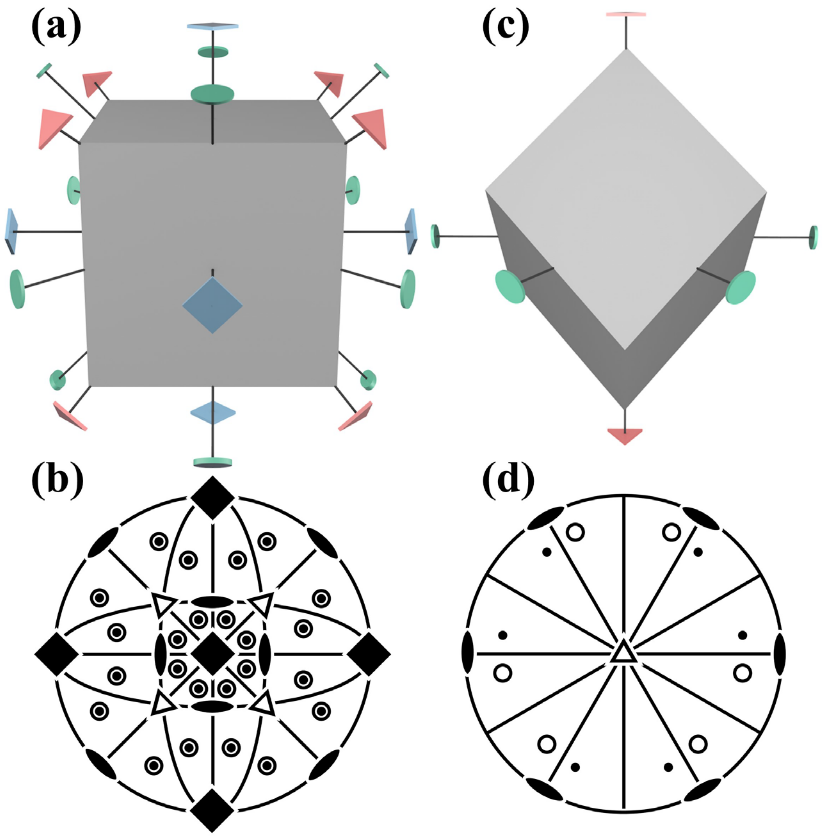

Fig. S6 Symmetry of point group *m*3*m* (O_h) and-3*m* (D_{3d}) (a) Characteristic diagram and (b) polar stereographic projection of point group *m*3*m* (O_h). (c) Characteristic diagram and (d) polar stereographic projection of point group -3*m* (D_{3d})

The symmetry of YIG with the space group *Ia*-3*d* (167) and the point group *m*3*m* (O_h) was analyzed. The characteristic diagram of the point group *m*3*m* (O_h) is shown in Fig. S6a. This point group possesses four threefold inversion axes S_3, three fourfold rotation axes C_4, six twofold rotation axes C_2, three mirrors σ parallel to C_4, six mirrors σ parallel to C_2, and one inversion center *I*. When the YIG lattice undergoes stress deformation within the (111) crystal plane, normally with equal lattice constants $a = b = c$, the S_3 axis along [111] remains unchanged, while the S_3 axes in other directions vanish. All C_2 axes not perpendicular to S_3 and mirrors σ not parallel to S_3 disappear,

leaving only one S_3 axis, three C_2 axes perpendicular to S_3, three mirrors σ perpendicular to C_2 and parallel to S_3. The inversion center *I* originally located at the body center remains unchanged. The point group *m*3*m* (O_h) transforms into -3*m* (D_{3d}), which remains a centrosymmetric point group. The characteristic diagram of the point group -3*m* (D_{3d}) is shown in Fig. S6c. To make the description more intuitive, the polar stereographic projections of the point groups *m*3*m* (O_h) and -3*m* (D_{3d}) are shown in Fig. S6b and S6d, respectively.

Supplementary References

1. Schreier, M., et al., Magnon, phonon, and electron temperature profiles and the spin Seebeck effect in magnetic insulator/normal metal hybrid structures. *Phys. Rev. B***, 88**, 094410 (2013).
2. Zhang, Q. G., Cao, B. Y., Zhang, X., Fujii, M. & Takahashi, K., Size effects on the thermal conductivity of polycrystalline platinum nanofilms. *J. Phys.: Condens. Matter***, 18**, 7937-7950 (2006).
3. Chen, Y. J. & Huang, S. Y., Light-induced thermal spin current. *Phys. Rev. B***, 99**, 094426 (2019).
4. Chen, Y. N., Ma, J. L. & Li, W., Understanding the thermal conductivity and Lorenz number in tungsten from first principles. *Phys. Rev. B***, 99**, 020305 (2019).
5. White, G. K. & Collocott, S. J., Heat capacity of reference materials: Cu and W. *J. Phys. Chem. Ref. Data***, 13**, 1251-1257 (1984).
6. Watjen, J. I., Bright, T. J., Zhang, Z. M., Muratore, C. & Voevodin, A. A., Spectral radiative properties of tungsten thin films in the infrared. *Int. J. Heat Mass Tran.***, 61**, 106-113 (2013).
7. Hofmeister, A. M., Thermal diffusivity of garnets at high temperature. *Phys. Chem. Minerals***, 33**, 45-62 (2006).
8. Agrawal, M., et al., Role of bulk-magnon transport in the temporal evolution of the longitudinal spin-Seebeck effect. *Phys. Rev. B***, 89**, 224414 (2014).
9. Guillot, M., Tcheou, F., Marchand, A., Feldmann, P. & Lagnier, R., Specific heat in erbium and yttrium iron garnet crystals. *Physik B Condens. Matter***, 44**, 53-57

(1981).

10. Angeles, F., et al., Interfacial thermal transport in spin caloritronic material systems. *Phys. Rev. Mater.*, **5**, 114403 (2021).

11. Wang, S. H., et al., Strongly extended diffusion length for the nonequilibrium magnons in $Y_3Fe_5O_{12}$ by photoexcitation. *Phys. Rev. Mater.*, **2**, 051401 (2018).

12. Wang, S. H., et al., Spin Seebeck effect and spin Hall magnetoresistance in the Pt/$Y_3Fe_5O_{12}$ heterostructure under laser-heating. *Chin. Phys. B*, **27**, 117201 (2018).

13. Ellsworth, D., et al., Photo-spin-voltaic effect. *Nat. Phys.*, **12**, 861-866 (2016).

14. Alexander, T., Subeshan, B. & Asmatulu, R., Modifying the figure of merit of thermoelectric materials with inclusions of porous structures. *Energy, Ecology and Environment*, **5**, 313-329 (2020).

15. Young, S. M., Zheng, F. & Rappe, A. M., Prediction of a linear spin bulk photovoltaic effect in antiferromagnets. *Phys. Rev. Lett.*, **110**, 057201 (2013).

16. Xiao, R. C., Shao, D. F., Li, Y. H. & Jiang, H., Spin photogalvanic effect in two-dimensional collinear antiferromagnets. *Npj Quantum Mater.*, **6**, 35 (2021).

17. Kim, K. W., Morimoto, T. & Nagaosa, N., Shift charge and spin photocurrents in Dirac surface states of topological insulator. *Phys. Rev. B*, **95**, 035134 (2017).

18. Xu, H., Wang, H., Zhou, J. & Li, J., Pure spin photocurrent in non-centrosymmetric crystals: bulk spin photovoltaic effect. *Nat. Commun.*, **12**, 4330 (2021).
