# Supplementary material for: Origin of the light-induced spin currents in heavy metal/magnetic insulator bilayers
Source: Nat Commun. 2024 May 22;15:4362. doi: 10.1038/s41467-024-48710-6 (PMC11111453; doi:10.1038/s41467-024-48710-6)
Supplement: Supplementary file 2 — Lasing Reporting Summary [file 41467_2024_48710_MOESM2_ESM.pdf]

nature research

Corresponding author(s): Lin Sun
NCOMMS-23-58837D

Lasing Reporting Summary

Nature Research wishes to improve the reproducibility of the work that we publish. This form is intended for publication with all accepted papers reporting claims of lasing and provides structure for consistency and transparency in reporting. Some list items might not apply to an individual manuscript, but all fields must be completed for clarity.

For further information on Nature Research policies, including our data availability policy, see Authors & Referees.

• Experimental design

Please check: are the following details reported in the manuscript?

1. Threshold

Plots of device output power versus pump power over a wide range of values indicating a clear threshold

- [ ] Yes
- [x] No

In this manuscript, the Xenon lamp and Laser were used to measure SPVE, in which no power threshold value exists.

2. Linewidth narrowing

Plots of spectral power density for the emission at pump powers below, around, and above the lasing threshold, indicating a clear linewidth narrowing at threshold

- [ ] Yes
- [x] No

In this manuscript, the Xenon lamp and Laser were used to measure SPVE, which is independent of linewidth narrowing.

Resolution of the spectrometer used to make spectral measurements

- [ ] Yes
- [x] No

The SPVE measurement is independent of the resolution of spectrometer.

3. Coherent emission

Measurements of the coherence and/or polarization of the emission

- [ ] Yes
- [x] No

The SPVE measurement is independent of coherence and polarization.

4. Beam spatial profile

Image and/or measurement of the spatial shape and profile of the emission, showing a well-defined beam above threshold

- [x] Yes
- [ ] No

Image of the spatial shape of the Xenon lamp were shown in Figure S1 of the Supplementary Information.

5. Operating conditions

Description of the laser and pumping conditions
Continuous-wave, pulsed, temperature of operation

- [x] Yes
- [ ] No

See Methods-SPVE measurements in the manuscript.

Threshold values provided as density values (e.g. W cm^{-2} or J cm^{-2}) taking into account the area of the device

- [x] Yes
- [ ] No

see Methods-SPVE measurements in the manuscript.

6. Alternative explanations

Reasoning as to why alternative explanations have been ruled out as responsible for the emission characteristics
e.g. amplified spontaneous, directional scattering; modification of fluorescence spectrum by the cavity

- [ ] Yes
- [x] No

This manuscript does not involves the emission characteristics of laser.

7. Theoretical analysis

Theoretical analysis that ensures that the experimental values measured are realistic and reasonable
e.g. laser threshold, linewidth, cavity gain-loss, efficiency

- [x] Yes
- [ ] No

See Methods-Heat transfer simulation model in manuscript and the Supplementary Information-section B.

8. Statistics

Number of devices fabricated and tested

- [x] Yes
- [ ] No

More than ten samples were fabricated and tested.

Statistical analysis of the device performance and lifetime (time to failure)

- [x] Yes
- [ ] No

The sample still shows a good SPVE signal after three months, indicating the stability of device performance.
